# A long-term field study on the effects of dietary exposure of clothianidin to varroosis-weakened honey bee colonies

**DOI:** 10.1007/s10646-018-1937-1

**Published:** 2018-05-03

**Authors:** Reinhold Siede, Marina D. Meixner, Maria T. Almanza, Ralf Schöning, Christian Maus, Ralph Büchler

**Affiliations:** 1 Landesbetrieb Landwirtschaft Hessen, Bieneninstitut, Erlenstr. 9, 35274 Kirchhain, Germany; 2 Bayer AG, Alfred-Nobel-Str. 50, 40789 Monheim, Germany

**Keywords:** Honey bee, Varroosis, Clothianidin, Interaction

## Abstract

Clothianidin is a commonly used systemic insecticide in seed treatments. Residues of clothianidin can occur in nectar and pollen as a result of within-plant-translocation. Foraging bees can collect contaminated nectar or pollen. Concerns have been brought forward that exposure to pesticide residues might affect colonies especially if they are weakened by varroosis. However, there are few scientific studies investigating such multiple-stressor scenarios in the context of the entire colony. To close this gapa field trial with 24 colonies was set up. The study design comprised four groups of six colonies each fed with uncontaminated sugar syrup ('C0'), or syrup spiked with 10 μg L^−1^ clothianidin ('C10'), 50 μg L^−1^ clothianidin ('C50') or 200 μg L^−1^ clothianidin ('C200'). C10 represented a residue concentration that may exceptionally occur and therefore a worst-case scenario, the higher dietary concentrations exceed and do not reflect fieldrealistic levels. A substantial load of 8 mites of Varroa destructor per ten gram bees in autumn was adjusted. The colonies were followed up for 328 days. The amount of brood and the strength of each colony were regularly assessed. Colony health, bee mortality, overwintering success, hive weights, and levels of in-hive residues were determined. Varroosis turned out to be the significant key factor for the endpoint colony strength. Clothianidin did not have a statistically significant impact on C0, C10 and C50 colonies. No statistical evidence was found for an interaction between varroosis andexposure to clothianidin.

## Introduction

Clothianidin is an insecticide with a high efficacy against a broad spectrum of sucking and biting insects (Elbert et al. 2008; Uneme 2011). Even relatively small amounts of the substance are sufficient to control pests of major agricultural crops (Elbert et al. 2008; Nauen et al. 2003). Owing to its systemic properties it is suitable for seed treatment products. As clothianidin has a relatively high water solubility of 0.340 g L^−1^ (at 20°C, pH 4–10, (Hazardous Substances Data Bank 2005), the active ingredient of the seed coating disperses to the surrounding soil moisture. The roots of the growing seedling take up the substance with the water flow. Clothianidin is easily transported within the xylem in acropetal direction to non-treated parts of the plants (Bonmatin et al. 2015; Maienfisch et al. 2001), so that insects feeding on the treated crops are exposed to the treatment. The amount taken up by the pests is sufficient to kill them during the early growth phase of the crop. Thus young seedlings that are sensitive to pest attack are protected. Treating seeds with clothianidin can help to prevent insect damage to the crops during the crucial phase of establishment. Clothianidin is used world-wide in a broad range of crops (Simon-Delso et al. 2015). In the European Union (EU), clothianidin was, for instance, applied to the seed of oilseed rape to protect the seedlings against rape flea beetle (*Psylliodes chrysocephalus*), cabbage root fly (*Delia radicum*), and diverse virus-transmitting aphids. Seed treatment with clothianidin in bee-attractive crops like oilseed rape or maize was a widespread farming practice in the EU until the restrictions in 2013 (EC 2013), and is still common in regions other than the EU. In the EU, clothianidin is currently not registered as a substance for seed application in oilseed rape, however, it is still in use as seed treatment for rape among others, for e.g., in Australia (https://portal.apvma.gov.au, accessed 05 December 2016), in Canada (http://www.hc-sc.gc.ca, accessed 05 December 2016), in USA, and in many other countries (Simon-Delso et al. 2015).

Honeybees might be exposed to clothianidin seed treatment in the first place through the uptake of systemic residues in nectar and pollen of seed-treated crops. This is considered the key route of exposure, although also other exposure scenarios have been documented or postulated (e.g., systemic residues in untreated plants, caused by soil residues, residues in water bodies, in guttation drops of seedlings of seed- or soil-treated plants (Blacquiere et al. 2012; Botias et al. 2015; Cresswell et al. 2012; Girolami et al. 2009; Main et al. 2014; Pohorecka et al. 2012; Schaafsma et al. 2015; Schmuck and Keppler 2003; Xu et al. 2016). The relevance of guttation as a potential exposure route for bees has been discussed intensively as residues in guttation fluids can reach toxic concentrations (Nikolakis et al. 2015; Reetz et al. 2011; Tapparo et al. 2011). However, there are no confirmed cases of bee intoxication as a result of collecting guttation fluids and experts consider the risk for the whole colony as low despite gaps in our knowledge (Pistorius et al. 2012; Thompson 2010). Another potential route of exposure was highlighted by an incident that occurred in Germany in 2008. During the drilling of clothianidin-treated maize seeds, dust from the seed coating was emitted with the planting machines’ exhaust air and deposited on nearby flowers. As a consequence, foraging insects, especially bees, were exposed and intoxicated (Forster 2009; Pistorius et al. 2010). Several thousand colonies belonging to ~ 700 beekeepers were affected. Experiments simulating the precipitation of dust from maize drilling confirmed that there is the potential of exposure to high levels of clothianidin lethal to bees under certain conditions (Krupke et al. 2012). However, it has been shown that exposure to dust can be minimized to levels that are not toxic to bees by the use of technical solutions on the levels of seed treatment quality and planter technology (Forster et al. 2012; Nikolakis et al. 2010). Several studies report detectable amounts of clothianidin residues in nectar and pollen of seed-treated oilseed rape. The residue levels are in the order of magnitude of a few µg kg^−1^; typically they are far below 5 µg kg^−1^ (Cutler and Scott-Dupree 2007; Cutler et al. 2014; Pohorecka et al. 2012; Rolke et al. 2016b; Woodcock et al. 2017).

Clothianidin is intrinsically highly toxic to honey bees. The LD_50_ for oral exposure was determined as 3.8 ng active substance per bee (Schmuck and Keppler 2003). However, the quantity of neonicotinoids collected by foragers from nectar and pollen of seed-treated crops is estimated to be far below lethal levels (Godfray et al. 2014). Therefore, an acute intoxication of the forager cannot be expected, but concerns have been raised that honey bee colonies might be damaged by a long-term exposure to small amounts of clothianidin. A number of studies have indicated that the substance may cause sublethal effects to honey bees. The compound can affect the bees’ immunity, (Di Prisco et al. 2013) or their home finding capacity (Fischer et al. 2014). An alteration of the foraging behavior is reported (Schneider et al. 2012). Thiamethoxam, another neonicotinoid, which may be metabolized to clothianidin was shown to reduce locomotion of bees (Charreton et al. 2015; Williamson et al. 2014), and to affect their orientation performance (Henry et al. 2012). However, it has to be taken into consideration that many of these studies found these effects at unrealistic exposure concentrations and/or otherwise unrealistic exposure conditions. Some studies address the topic with experimental colonies under field-like conditions (Cutler et al. 2014; Pilling et al. 2013; Pohorecka et al. 2012; Sandrock et al. 2014) but the impact on colonies under farming standard conditions is less clear. However, in studies where honey bee colonies were exposed to treated crops in a field-realistic scenario (European Food Safety Authority (efsa) 2013);, (Cutler and Scott-Dupree 2007; Cutler et al. 2014; Pohorecka et al. 2012; Rolke et al. 2016a; Rundlof et al. 2015), no adverse effects were found.

Generally, honey bee colonies are regularly infested by various pathogens and parasites (Genersch et al. 2010; Guzmán-Novoa et al. 2010). One of the most important is the parasitic *Varroa destructor* mite, which is one of the main causes of colony losses in the northern hemisphere (Neumann and Carreck 2010). Besides direct damage by mites and by viruses they are transmitting, parasitization is thought to render colonies more susceptible to diseases or to abiotic stressors as pesticides (Doublet et al. 2015). Therefore, the experiment was undertaken with the objective to address the following two questions: (a) Are honey bee colonies harmed by a long-lasting exposure to sublethal dietary concentrations of clothianidin? (b) How do colonies perform under combined stress from a substantial *V. destructor* infestation and exposure to clothianidin?

## Materials and methods

### Experimental design and colony management

At the beginning of June 2014, 24 experimental colonies of *Apis mellifera carnica* (L.) were established from shook swarms consisting of 1.5 kg bees. Young sister queens, mated at the mating yard “Gehlberg”, Germany, were added to the swarms. The swarms were randomly distributed to four groups of six hives each. Group C0 was the control, which was not exposed to clothianidin. Group C10, C50, and C200 were treatment groups, orally exposed to clothianidin by feeding spiked syrup with nominal concentrations of 10 µg L^−1^, 50 µg L^−1^, and 200 µg L^−1^. Throughout the experiment, each syrup feeding was done with the ready-to-use formulation Ambrosia^®^ (Nordzucker AG, 38100 Braunschweig, Germany). Ambrosia^®^ consists of a mixture of glucose, fructose, and sucrose. Its nutritive value corresponds to ~ 0.73 kg sucrose per L syrup. The swarms were encased in magazine hives with eight Zander wax foundations and two drawn Zander combs. The colonies were located at a bee yard of Landesbetrieb Landwirtschaft Hessen in Central Germany. The site was mainly surrounded by arable crop land. No major nectar sources were within a radius of 3 km from the experimental site during the exposure period. Colonies of each group were arranged in blocks with distances of ~ 50 m between groups. From the beginning of the experiment in June 2014 to 13 August 2014 (69 DAI = days after initiation of the experiment) each colony in a treatment group was provided on four occasions with 5 L of spiked syrup (C10, C50, C200) or uncontaminated (C0) syrup. Feeding dates were 5 June 2014; 1 July 2014; 24 July 2014, and 13 August 2014. In September, each colony was fed twice with 5 L syrup without clothianidin. In October, according to the colony-specific demands a further 2–5 L uncontaminated syrup were fed. Colonies of the C200 group received only two feedings of 5 L, each following the exposure period, as they were too weak to consume the food from the feeder. Colonies that lost their queen during summer were re-queened with a sister queen from the original cohort. At the beginning of April 2015, a drone comb was added to all colonies.

Colonies were treated for Varroosis when a predefined threshold was exceeded. The threshold values were 0.2 mites g^−1^ bees for the shook swarms, 0.1 mites g^−1^ bees in mid July (14.07.; 39 DAI) and 0.8 mites g^−1^ bees in mid September (23.09.; 110 DAI). Colonies from heavily infested shook swarms were treated with 15% lactic acid on 12 June 2014. Colonies exceeding the thresholds in mid July or in mid September were treated with 2–4 strips of flumethrin (Bayvarol^®^–Bayer Vital GmbH, 51368 Leverkusen, Germany) according to colony size. In preparation for winter, all colonies were treated with 25–50 ml coumaphos (Perizin^®^–Bayer Vital GmbH, 51368 Leverkusen, Germany) according to colony size.

At the end of the experiment (29.04.15; 328 DAI) all colonies were killed (details see section ‘Precise determination of colony strength at the end of the study’). After that the amount of brood was measured and the number of bees counted.

### Exposure to clothianidin

Clothianidin as a primary reference substance with a certified purity of 99.4 % w w^−1^ was obtained from Bayer AG. Stock solution I was prepared by dissolving 50 mg clothianidin in de-mineralized water at a final volume of 0.5 L (100 mg L^−1^). A 50 ml aliquot of stock solution I was diluted with 0.45 L de-mineralized water to obtain stock solution II (10 mg L^−1)^. From stock solution I, 64 ml were added to 0.936 L water, shaken for 1 h in the dark, and with the help of a stirrer thoroughly mixed in a barrel containing 31 L syrup, resulting in a concentration of 200 µg L^−1^. The syrup containing 50 µg clothianidin L^−1^ was prepared accordingly by adding 1 L of a mixture of 160 ml of stock solution II and 0.840 L water. Syrup containing 10 µg clothianidin L^−1^was prepared by adding a mixture of 32 ml stock solution II and 0.968 L water to 31 L syrup. For the control group 1 L water was added to the syrup. Fresh syrup batches were prepared a few days before each feeding date. The residue analytical laboratories of Bayer AG determined the actual concentrations with a LOQ = 0.001 mg kg^−1^ and a limit of detection (LOD) of 0.0003 mg kg^−1^. Clothianidin content in the syrup of the controls (C0) were below the LOD, in the syrups of group C10, 7.4 µg kg^−1^ ( = 10.36 µg L^−1^), of group C50, 38 µg kg^−1^ ( = 53.2 µg L^−1^), and of group C200, 147 µg kg^−1^ ( = 205.8 µg L^−1^). The feeding scheme is indicated above in the section entitled “Experimental Design and colony management”.

### Residue analysis

Samples of 10 g bee bread, 15 g stored syrup, 10 larvae, 25 adult hive bees, and 25 adult forager bees per colony were collected on DAI 69, 112, 286, and 321. To avoid risks of contamination, samples were collected with disposable gloves and single-use plastic cutlery. All samples were transported and stored in disposable plastic sample containers. Immediately after removal from the colonies, the samples were placed on dry ice. Samples were labeled with blind numbers and stored at −20°C. Then they were shipped in dry ice to the analytical laboratories of Bayer AG, where they were analyzed for residues of clothianidin and its metabolites TZNG and TZMU. The chemical analyses followed standard procedures (Schoening 2001).

### Health parameters of the colonies

The colonies were checked for their rate of infestation with the parasitic mite *V. destructor*. Mites were counted at the beginning of the experiment, and on DAI 39, 68, 110, and 284. Sampling and counting followed the protocol of the COLOSS BEEBOOK according to the soapy water method (Dietemann et al. 2013). Adult bees were checked for infection with *Nosema* spp. Approximately 40 g of bees (corresponding to ~ 400 bees) were collected from a peripheral frame in July, September, and March with the intention to minimize the effect of sampling on the rather small-sized colonies. From each sample, 60 bees were randomly selected. The bees’ abdomen were removed and macerated in plastic bags with 6 ml water with the help of a laboratory blender (Stomacher^®^, Seward, Lt. West Sussex, BN14 8HQ, UK). Aliquots of the resulting suspension were microscopically checked for *Nosema* spores, which were counted with a Bürker hemacytometer. The viral load of the colonies was analyzed at the end of the experiment according to previously published PCR-based protocols (Cox-Foster et al. 2007; Siede et al. 2008).

### Performance of colonies

The strength of the colonies was estimated according to Liebefelder method on three occasions before winter (26, 68, 110 DAI) and twice after overwintering (284, 320 DAI) (Imdorf et al. 1987). During estimation of the colonies the entrances were closed to prevent robbery. The number of bees and number of brood cells were assessed by three experienced staff members of Bieneninstitut Kirchhain. All three assessors were trained with the same material and verified their estimates among each other prior to the experiment to achieve equivalent results. The weight of the colonies was recorded seven times (26, 68, 89, 112, 124, 284, and 320 DAI) with an electronic hive-scale (Bosche, Wägetechnik, Damme, Germany, model TWI). Net weights adjusted for the weight of hive material were used for the statistical comparison of the four treatment groups.

### Honey bee mortality

Adult bee mortality was measured with dead bee traps. These were two nested mesh cages. The lower wire box were 46 cm large, 5 cm high, and 10 cm deep and had a mesh size of 4 mm. The overlapping upper part with the same dimensions was made from wire with a mesh size of 8 mm. The cages were fixed in front of the hive in a manner that the complete entrances were covered by the wire boxes. All outgoing bees had to pass through the boxes. Dead bees could be collected from the lower part of the boxes. Two days after the administration of the clothianidin spiked syrup, all dead bees were removed from the traps and were counted. In the region of the study, there is no flight activity in winter. Therefore, dead bees drop out of the winter cluster and accumulate on the bottom board of the hives during winter The number of dead bees on the bottom boards were counted in February (257 DAI) before the cleansing flight and well before discarding the dead bees by nest mates. Colony mortality was defined as the removal date from the yard. C200 colonies were removed when the bees occupied not more than a single comb. During winter the colonies were checked once a week from 3 November 2014 till 26 February 2015. For inspection, the covers of the hives were lifted carefully. Colonies were removed from the yard when less than three beeways between the combs were occupied by bees.

### Precise determination of colony strength at the end of the study

At the end of the trial (328 DAI), the colonies were weighed. The bees were brushed off the combs with a box-shaped device (‘Kehrfix’®, Carl Fritz Imkertechnik GmbH & Co. KG, 97638 Mellrichstadt, Germany) in the early morning before flight activity. The bees were re-collected in wire mesh boxes. The boxes were placed in two-chamber bee hives, which were firmly closed up. Bees were killed by fumes from burning 20 g sulfur stripes in the upper chamber. The exact numbers of bees of each colony were counted manually. The area of brood in the frames of the hives was recorded with a marker to transparent sheets, scanned and the area of recorded brood measured using the open-source digital imaging software imageJ (Version 1.51n) (Abràmoff et al. 2004).

### Statistical analysis

Data were tested with the procedure linear mixed models in spss v. 20. Number of bees, number of brood cells, percentage of uncapped brood and the net weights of the colonies were log-transformed. Number of *V. destructor* per 10 g bees were log (x + 1) transformed and used as co-variant. An autoregressive covariance type of first order was assumed. DAI was used to define the repeated measures. DAI, treatment, and infestation with *V. destructor* as well as the interactions between treatment and DAI and between *V. destructor* and treatment were considered as fixed effects. Owing to the rapid breakdown of the colonies of the C200 group these were not considered in the statistical model. Number of bees in the dead bee traps attached to the entrance of colonies were aggregated by the area under curve (AUC) approach. analysis of variance (ANOVA) was performed with the AUC values and with the number of dead winter bees. Time periods of survival were analyzed using the Kaplan–Meier method (Bühl 2012). Survival time of colonies, which survived till the end of the experiment, was estimated as 328 days. Differences between groups were tested for significance with the log-rank test (Mantel–Cox test). Means of residues in Table 1 and the online resource 1 were calculated from detection above the limit of quantification (LOQ). Spearman two sided rank correlation coefficients were calculated between the numbers of bees at the end of the trial, the numbers of brood cells at the end of the trial, the means of the residues of clothianidin (parent compound) in the stored syrup and the mean infestation rate of *V. destructor*. For that purpose residue values of clothianidin below LOD were replaced by the half of LOD ( = 0.15 µg kg^−1^) and in case of values between LOD and LOQ by 0.5 µg kg^−1^ (US Environmental Protection Agency (EPA) 2000; US Environmental Protection Agency (EPA) 2006).

**Table 1:** Means of residues of clothianidin (µg kg^−1^)

		Days after initiation			
Group	Matrix	69	112	286	321
C0	Bee bread	<LOQ	<LOD	<LOD	<LOD
	Forager bees	<LOD	<LOD	<LOD	<LOD
	Food	1.4	<LOD	1.7	<LOQ
	Hive bees	<LOQ	<LOD	<LOD	<LOD
	Larvae	<LOD	<LOD	<LOD	<LOD
C10	Bee bread	1.8	<LOQ	1.6	1.5
	Forager bees	<LOD	<LOD	>LOQ	<LOD
	Food	7.3	3.9	4.2	4.8
	Hive bees	2.4	<LOD	<LOQ	<LOD
	Larvae	1,7	<LOD	<LOD	<LOD
C50	Bee bread	8.1	1.9	2.8	4.7
	Forager bees	1.3	<LOD	2.3	<LOQ
	Food	27.4	11.2	22.8	22.7
	Hive bees	5.2	2.0	2.5	<LOQ
	Larvae	1.7	1.2	1.4	<LOQ

LOQ = 1 µg kg^−1^, LOD = 0.3 µg kg^−1^, means per row were calculated from values > LOQ. Residues were determined in the hive matrices bee bread and food and in the three bee states (larvae, hive bees, and foragers) of controls (group C0) and the two treatment groups (C10 and C50)

## Results

### Residues

Clothianidin residues were found in bee bread samples of the C10 and C50 group (see Table 1 and additional data given in the online resource). The mean clothianidin residue in the C10 bee bread sample was 1.8 µg kg^−1^ at 69 DAI, < LOQ at 112 DAI, 1.6 µg kg^−1^ at 286 DAI, and 1.5 µg kg^−1^ at 321 DAI. Clothianidin residues in bee bread of the C50 samples ranged between 1.9 µg kg^−1^and 8.1 µg kg^−1^. Only one sample of C0 bee bread was contaminated with a residue content below LOQ on 69 DAI. C200 colonies did not collect pollen. Therefore, residue analysis of bee bread of C200 colonies was not possible. In mid-August at 69 DAI stored syrup was contaminated with 1.4 µg kg^−1^ for C0 colonies, 7.3 µg kg^−1^ for C10 colonies, 27.4 µg kg^−1^ for C50 colonies and 108.6 µg kg^−1^ for the C200 group. The contents decreased in autumn (112 DAI, C0: < LOD; C10: 3.9 µg µg kg^−1^, C50: 11.2 µg kg^−1^) and after winter leveled off at group characteristic concentrations (C0: ≤ 1.7 µg kg^−1^, C10 ≤ 4.8 µg kg^−1^, and C50 ≤ 22.8 µg kg^−1^). The highest concentrations occurred in stored syrup with 22.9 µg kg^−1^, which is the mean across the four sampling dates and all groups. The other analyzed matrices contained much lower residues of clothianidin. Hive bees were loaded with a mean of 7.1 µg kg^−1^, larvae with a mean 2.7 µg kg^−1^, and forager bees with a mean of 2.1 µg kg^−1^.

Quantifiable amounts of the metabolite TZNG were found in 14 samples out of 324 analyzed samples where 8 samples belonged to group C200 and 6 samples belonged to group C50. From the 14 samples with detects of TZNG, two were hive bees and 12 samples were stored syrup samples. Out of the 324 analyzed samples, the metabolite TZMU was quantifiable in 9 samples only. Five of them belonged to group C200 in the matrices hive bees (four detections, mean: 2.28 µg kg^−1^; SD:2.32 µg kg^−1^) and stored syrup (one detection, 4.85 µg kg^−1^) and four to group C50 in stored syrup (mean: 1075 µg kg^−1^; SD = 0.15 µg kg^−1^).

### Loads with pest and pathogens

**Fig. 1:**
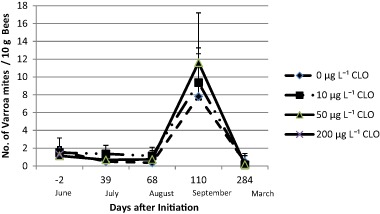
*Varroa destructor* infestation rate as a function of time and the exposure to clothianidin. error bars: + 1 SD; For group C200 there is only one data point at −2 DAI as these colonies dwindled rapidly. CLO = clothianidin

All colonies were substantially infested with *V. destructor* (see Fig. 1). At the beginning the mean loads of the artificial swarms were 1.67 mites/10 g bees for the C0 group, 1.5 mites/10 g bees for the C10 group, 1.14 mites/10 g bees for the C50 group, and 1.28 mites/10 g bees for the C200 group. Differences were not significant (*p* = 0.818, ANOVA data log(x + 1) transformed). As the colonies were treated for Varroa only when predefined thresholds of infestation were exceeded (see material and methods) the infestation rates during the summer remained more or less constant. The respective values for the important period of winter bee production in July were 0.5 mites/10 g bees for C0, 1.37 for C10 and 0.70 for C50. There are no values for the C200 colonies as they were already collapsed at that time. The respective values in August were 0.37, 1.14, and 0.74 mites/10 g bees. In September, (110 DAI) there was a sharp increase to 7.82 mites/10 g bees for the C0 group, 9.37 mites/10 g bees for the C10 group, and 11.62 mites/10 g bees for the C50 group, The means of infestation in number mites per 10 g bees calculated over all five sampling dates were 2.22 for C0 colonies (*N* = 29, SD = 3.82), 2.99 for the C10 colonies (*N* = 27; SD = 4.95) and 2.97 for the C50 colonies (*N* = 29, SD = 6.11). For the C200 colonies, no data could be measured due to the rapid collapse of the colonies following exposure to the treatment. After the application of coumaphos in winter a mean of 186 mites were collected from the C0 colonies. (SD = 185; *N* = 6). From the C10 colonies, 700 mites dropped (SD = 637, *N* = 5) vs. 319 mites from C50 colonies (SD = 417, *N* = 5). There was no statistical difference in the number of Varroa mites collected in each of the treatment groups (*p* = 0.428, Kruskal–Wallis Test). At the end of the experiment the mite-vectored acute bee paralysis virus was not detected in the analyzed samples. Deformed wing virus was found in eight out of 13 colonies (Table 2). *Nosema* spore levels were well below those that would result in beekeeper concern (Table 2).

**Table 2:** Mean *Nosema* spp. spore loads and occurrence of viruses in C0, C10, and C50 colonies

	No. of spores of *Nosema* spp.			Viruses (number of positive detections)	
Group	14.07.14 39 DAI	23.09.14 110 DAI	16.03.15 284 DAI	ABPV 328 DAI	DWV 328 DAI
C0	0	56,667	10,000	0 (out of 5)	3 (out of 5)
C10	48,333	0	0	0 (out of 3)	2 (out of 3)
C50	0	463,333	0	0 (out of 5)	3 (out of 5)

ABPV: Acute Bee Paralysis Virus; DWV: Deformed Wing Virus. Group C200 is not included because C200 colonies were very weak or died prior to the assessment time

### Bee mortality

The number of bees in dead bee traps were determined 2 days after feeding at 2 DAI, 28 DAI, 51 DAI, and 71 DAI with an overall mean of 28 dead bees for colonies of the C0 group, 37 for C10, and 30 for C50. Differences between the groups were not statistically significant (*p* = 0.68, ANOVA of the AUC values). Colonies of the C200 group had only three dead bees in the trap (mean of 2 DAI, 28 DAI, and 51 DAI). The majority of the dead bees of C200 colonies were found inside the hive. The numbers of dead bees collected in February from the bottom boards of the hives were not statistically different between groups (*p* = 0.236, ANOVA). On the average C0 colonies had 1720 dead bees (*N* = 6, SD = 666), C10 colonies 2076 (*N* = 3, SD = 1309) and C50 colonies had 1110 dead bees (*N* = 5, SD = 469) on the bottom boards.

### Survival of the colonies

The colonies of the C200 group that were exposed to an acutely toxic dietary concentration of clothianidin lost the largest proportion of their bees within 2 months. Estimated mean period of colony survival was 316 days for C0, 281 days for C10, 304 days for C50, and 55 days for C200. (see Table 3). Differences of the survival period between C0, C10, and C50 were not statistically significant (*p* = 0.368; Mantel–Cox test). During the winter 1 C0, 3 C10, and 1 C50 colony (colonies) did not survive. No obvious effects on queen survival were observed. Only one C10 colony had lost its queen, which was compensated by re-queening. Table 4

**Table 3:** Period of survival of the test colonies in (d), Kaplan–Meyer analysis, differences between group C0, C10, and C50 were not significant (log-rang test, Mantel–Cox test: *p* = 0.368)

Group	Mean estimate	St. error	95% confidence interval	
			Lower bound	Upper bound
C0	316,167	10,802	294,994	337,339
C10	280,833	21,483	238,726	322,940
C50	304,500	21,452	262,453	346,547
C200	54,667	8433	38,138	71,195
Overall	239,042	23,399	193,180	284,903

**Table 4:** Number of colonies (*N*) that were estimated on each date of assessment

Group	01 July 14	12 August 14	23 September 14	16 March 15	21 April 15	29 April 15
DAI	26	68	110	284	320	328
C0	6	6	6	5	5	5
C10	6	6	6	3	3	3
C50	6	6	6	5	5	5
C200	5	2	0	0	0	0

DAI: days after initiation

Each group started with six colonies at the beginning of the experiment. In case of *N*<6, colonies were so weak that they had been removed from experimental yard to avoid robbery

### Parameters of colony performance and health

**Fig. 2:**
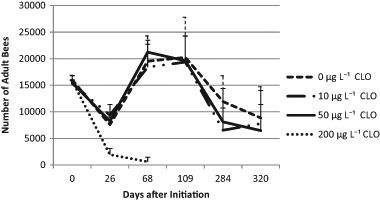
Numbers of adult bees per colony (colony strength) assessed according to the Liebefeld method as a function of time and the exposure to clothianidin; error bars: + 1 standard deviation (SD). At DAI 0 the numbers of bees were converted from the bee weight of the shook swarms with the assumption that 1 kg bees corresponds to 10,000 bees. During the period of DAI 68 to DAI 320 there was no significant difference between the groups C0, C10, and C50 (*p* = 0.708). CLO = clothianidin

**Fig. 3:**
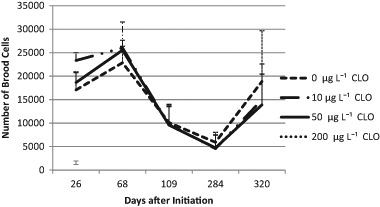
Number of brood cells per colony as a function of time and the exposure to clothianidin; error bars: + 1 SD. Treatment groups C10 and C50 were not significantly different from group C0 (*p* = 0.593). CLO = clothianidin

**Fig. 4:**
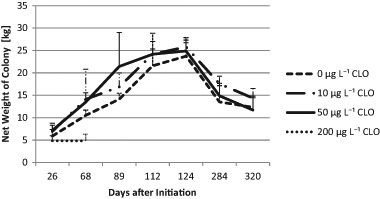
Net weight of the colonies as a function of time and the exposure to clothianidin; error bars: + 1 SD, Weights were corrected for addition or removal of the weights of hive material. CLO = clothianidin

The parameters colony strength (number of adult bees per colony), number of brood cells, and colony weight were measured. Colonies of the C200 group suffered from a massive loss of bees following exposure to the treatment. The other treatment groups were not significantly affected by exposure to clothianidin at the given concentrations based on the parameters measured. The strength of the colonies was not influenced by the exposure to clothianidin (*p* = 0.536; see Fig. 2). On average, C0 colonies had 14,246 bees (*N*_obs_ = number of observations = 26; SD = 7247) vs. 13,582 bees of the C10 colonies (*N*_obs_ = 24; SD = 6837) and 13,123 bees of the C50 colonies (*N*_obs_ = 28, SD = 7502) and 1817 bees of the C200 colonies (*N*_obs_ = 6; SD = 679). Interactions between exposure to clothianidin and time were not significant (*p* = 0.163). The infestation with *V. destructor* was a significant factor related to colony strength (*p* = 0.004). Interaction between *V. destructor* and treatment was not significant (*p* = 0.463). Exposure to clothianidin had no significant influence to the numbers of brood cells (Fig. 3, *p* = 0.865). No significant interactions were found between time and exposure (*p* = 0.700). *V. destructor* played a significant role for the production of brood cells (*p* < 0.001). The interaction between *V. destructor* and treatment was not significant for brood (*p* = 0.977). The mean values for brood cell numbers were 14,869 in the C0 colonies (*N* = 26; SD = 7089) vs. 17,201 in the C10 colonies (*N* = 24, SD = 9136) and 14,845 in the C50 colonies (*N* = 28, SD = 8119) and 1150 in the C200 colonies (*N* = 6, SD = 679). The percentage of uncapped brood of C0, C10, and C50 was not dependent on the treatment (*p* = 0.797), nor on the interactions of time and treatment (*p* = 0.390). The means were 39% (C0, *N* = 27, SD = 12.225), 37% (C10; *N* = 24; SD = 16.4), 36% (C50; *N* = 28; SD = 14.6) and 85% (C200, *N* = 6; SD = 14.6). With respect to the net weights of the colonies, there was a trend of a clothianidin induced increase (*p* = 0.089). Interactions between treatment and time were not significant (*p* = 0.711) but again, *V. destructor* had a significant negative impact on colony weight (*p* = 0.005). Interactions between *V. destructor* and treatment were not significant (*p* = 0.402). Colonies of the C0 group (14.81 kg, *N* = 38; SD = 656) were lighter than the exposed colonies (see Fig. 4). The respective mean values were 17.312 kg for C10 colonies (*N* = 36; SD = 7.17), 17.65 kg for the C50 colonies (*N* = 40; SD = 7.71) and 4.84 kg for C200 colonies (*N* = 10, SD = 1.19).

### Colony strength at the end of the experiment

The surviving colonies of all treatment groups (five C0 colonies, three C10 colonies, and five C50 colonies) were killed at 328 DAI. The mean values were 17,645 bees and 23,386 brood cells for C0 colonies, (*N* = 5) vs. 13,141 bees and 26,015 brood cells for C10 colonies (*N* = 3), and 12,615 bees and 19,654 brood cells for C50 colonies (*N* = 5) (see Table 5). Both, the smallest colony with 4,950 bees and the largest colony with 28,737 bees belonged to the C50 group. The differences between the groups were not statistically significant (*p* = 0.601). A significant correlation was found between the colony strength at the end of the experiment and the infestation rate of *V. destructor* (see Table 6, Rho = −0.665; *p* = 0.013, Spearman rang correlation). Residues levels were not linked to any of the parameters (*p* < 0.565, Table 6, Spearman rank correlation). The C0 colonies had 4943 drone brood cells (SD = 3163). C10 colonies had 3906 drone cells (SD = 2818) and C50 colonies 3610 drone cells (SD = 2517, all values are means). The differences were not statistically significant (*p* = 0.752).

**Table 5:** Counted numbers of adult bees and brood cells per colony at the end of the trial in April 2015, 328 days after the set up of the experiment (in parentheses: *N*: number of colonies); differences between the groups were not significant (ANOVA; SD: standard deviation)

	C0 (5)	C10 (3)	C50 (5)	*p*
Bees	17,645	13,141	12,615	0.601
SD	6320	8457	9659	
Brood	23,386	26,015	19,654	0.755
SD	14,221	13,254	7856

**Table 6:** Spearman rank correlation of the variables *V. destructor* (mean infestation rate), residues of clothianidin in the stored syrup (65 measurements), and the amount of bees and of brood cells at the end of the experiment after sacrificing the colonies

	Bees	Brood	*V. destructor* ^a^	Residues^b^
Bees	1	0.621*	−0.665*	0.038
		0.024	0.013	0.901
		13	13	13
Brood		1	−0.297	0.176
			0.325	0.566
			13	13
*V. destructor*			1	−0.046
				0.791
				18
Residues				1

^a^Mean rate of infestation

^b^Mean residues of the parent compound in stored food

Each line contains the Spearman’s rank correlation coefficient, its significance level and the sample size

Statistically significant correlations (p < 0.05) are indicated by bold letters

## Discussion

Honey bee colonies were fed with clothianidin-contaminated sugar syrup. The nominal concentrations were 0 µg L^−1^, 10 µg L^−1^ 50 µg L^−1^, and 200 µg L^−1^. The colonies were followed until DAI 328. As expected, the C200 colonies, which were exposed to the unrealistically high concentration of 200 µg L^−1^, dwindled within 2 months. The survival rates were 85% for the C0 group, 85% for the C50 group, and 50% for the C10 colonies. Exposed colonies had more clothianidin residues in in-hive matrices, more *Varroa* mites and tended to have a reduced colony strength, less brood, and a shorter mean survival period than the control colonies. However, differences were not statistically significant, and none of the endpoints was dependent on exposure. Moreover, there was no clear dose-response relationship given for any differences between the groups. Significant variables were time and infestation with *V. destructor*. Interactions between time and treatment were never statistically significant indicating that statistically there was no delayed effect of the treatment on the endpoints. *V. destructor* infestation significantly influenced the colony strength, number of brood cells and colony weight. As the interactions between *V. destructor* and the treatment were not significant we did not observe indications for synergism between both stressors. At the end of April in the year following exposure to the test substance, colony strengths were determined precisely. At that time exposed colonies had ~ 26% (C10) or 29% (C50) less bees than C0 colonies, but again, the differences were not statistically significant. In absolute numbers, C10 colonies had a mean of 4505 bees and C50 colonies had 5030 bees less than control colonies. Correlative evidence was found for a significant effect of *V. destructor* but not for an effect of the residues of clothianidin.

Low statistical power is a common phenomenon of field assays with honey bee colonies (Cresswell 2011; Pilling et al. 2013). The number of hives, which would be required to conduct an optimal statistical evaluation, can amount to several hundred of hives, especially if the study deals with low effect sizes (EFSA Panel on Plant Protection Products and their Residues (PPR) 2012). These numbers are likely to exceed the number of colonies, which can technically and logistically be handled in the field. Current valid regulatory guidelines demand for three replicates, only, with the aim to allow statistical testing (OEPP/EPPO 2010). However, this has been criticized as such a low number of replicates does not allow for the detection of small effects where a ‘small effect’ has been defined as an increase of daily forager losses about the factor 2 compared with untreated controls (EFSA Panel on Plant Protection Products and their Residues (PPR) 2012). Regarding our study, inferential statistics was performed but it cannot be excluded, that possible effects of the insecticide remained unnoticed due to the relatively low sample size. A recent large correlative study of thousands of fields cultivated with imidacloprid treated oilseed rape claimed to work out subtle effects of pesticides on bee colonies, given the availability of huge numbers of data sets (Budge et al. 2015); however, unfortunately the study failed to investigate a larger set of factors known to likewise influence bee health.

Without doubt, the infestation with *V. destructor* was a significant driver for reduced colony performance in general. In our study, there was a significant correlation between the level of parasitization and the strength of colonies at the end of the experiment. During the period of the experiment, *V. destructor* was a significant factor for the parameters colony strength, number of brood cells, net weights of the colonies and proportion of uncapped brood. This study intended to investigate a possible influence of clothianidin on colonies, which are affected by *V. destructor*, as devitalization of colonies by varroosis is a common situation in real beekeeping. Therefore, we deliberately opted for a soft mite control concept. Substantial numbers of *V destructor* dropped from the colonies after the winter treatment, which demonstrated that the colonies were really infested with *V. destructor*.

This study simulated a worst case scenario. Exposure levels of clothianidin were at the high end, even in the lower exposure level group, or even strongly exaggerated. Bees were exposed to contaminated food for a long period of time. Both, the levels of residues as well as the long duration exceeded a realistic exposure in the field. In nectar/pollen of seed-treated oilseed rape clothianidin residues significantly below 10 µg kg^−1^ are typically found (Blacquiere et al. 2012; Rolke et al. 2016b; Schmuck and Keppler 2003). As colonies usually do not forage exclusively on one individual crop, a dilution of contaminated nectar can furthermore be assumed. In reality the time of exposure would also be drastically shorter, as at the end of the season the beekeeper normally removes the honey from the hive and replaces it by syrup for overwintering. In this study, no food was removed nor a major nectar flow diluted the clothianidin-contaminated food of the experimental colonies. The recovery rates (see Table 1) suggest that the dilution factor was not higher than two, whereas metabolization and clearance of clothianidin are not taken into account. The data prove the very high exposure of the colonies. Interestingly also on the level of the individual bees the exposure exceeded even the values of the laboratory derived oral LD50 values of 3.8 ng (Schmuck and Keppler 2003). Assuming a daily sugar uptake of 18 mg per bee ( = 24.7 µl of syrup), (Blacquière and van der Steen 2017), a life expectancy of 28 days and a real mean clothianidin content of 5 µg kg^−1^( = 0.007 ng µL^−1^; C10), 21 µg kg^−1^( = 0.029 ng µL^−1^; C50), and 108 µg kg^−1^( = 0.151 ng µL^−1^; C200, see Table 1) each bee was exposed to a total dose of 4.88 ng, (C10), 20.32 ng (C50), and 104.39 ng (C200). Although all of them are above the LD50 there was no elevated mortality except for C200 colonies. A similar phenomenom had been observed for the neonicotinoid imidacloprid. (Blacquière and van der Steen 2017) explained that phenomenon by detoxification or elimination of the substance at a rate of 2.2 ng per 24 h (Cresswell et al. 2014). LD50 are determined by exposing bees to the doses at a single blow. As the bees of the field study were exposed to high amounts of clothianidin continuously their detoxification system has more time to get off the substances. Supposing that the clothianidin elimination is similar to the imidacloprid clearance each bee has the potential to eliminate a total of 61.6 ng, which is above the doses absorbed by the bees.

The no observable effect concentration (NOEC) of clothianidin for individual worker bees was determined in a 10 days lasting feeding study in the laboratory. A NOEC of 10 µg L^−1^ or 8.13 µg kg^−1^ was found (European Food Safety Authority (efsa) 2013). Thus, short- or mid-term effects were unlikely to be seen in the C10 colonies, and as no significant effect for the whole period of the experiment was observed, it can be concluded that there was likewise no cumulative effects. C50 colonies were confronted with a nominal dietary concentration of 50 µg L^−1^ and a verified concentration of approx. 20 µg kg^−1^. No indications neither for short-term nor cumulative effects were observed. Modeling time effects of imidacloprid, another neonicotinoid, on honey bees, predicted a marked increase of the sensitivity with time (Rondeau et al. 2014). The authors of this study hypothesized that despite low exposure concentrations there might be a huge biological damage, as neonicotinoid substances allegedly would bind irreversibly to neural receptors. Our data do not support such assumptions for clothianidin. At least under field conditions, we do not see evidence for a time-enhanced effect of clothianidin at the colony level.

Colonies of eusocial insects have mechanisms to compensate for disturbances by external factors. A reduced lifespan of specific individuals, which might have been more exposed to a toxicant could be out-balanced by an increase of life expectancy of other individuals. If higher clothianidin concentrations affected the success of brood rearing it could be compensated by an increase of the lifespan of the adults or by an increase of the investment in brood. The quotients of adult bees to bee brood were 1.2 adult bees per brood cell for C0, 1.1. for C10, and 1.1. for C50 colonies. However, differences between the control and the C10 and C50 colonies were not statistically significant neither for the parameters number adult bees nor number brood cells nor for the quotient between both parameters. There is no evidence for a clothianidin induced effect nor for a compensative reaction. If colonies react in that matter the capacity for compensation of the strongly overexposed C200 colonies was obviously exhausted.

The findings of our study are in agreement with quite a large body of field assays. Most of the experimental studies did not find significant effects of clothianidin residues in nectar or pollen from seed treatment (Cutler and Scott-Dupree 2007; Cutler et al. 2014; Rolke et al. 2016a; Rundlof et al. 2015) nor from treatment with thiamethoxam, a neonicotinoid, which can be metabolized to clothianidin (Pilling et al. 2013). Colony-feeding experiments are reported with small colonies, which were not affected by high concentrations up to 20 µg clothianidin kg^−1^ (Schmuck and Keppler 2003). To our knowledge there is only one investigation that reports effects of clothianidin on honey bee colonies in the field (Sandrock et al. 2014). The authors of this study exposed bees by in-hive feeding of contaminated pollen pasties. Effects on the number of bees were observed at two points of time, shortly after application of the substances, and a delayed effect was observed a year after the application of the compounds. However, in contrast to the studies cited before, the exposure of bee colonies in Sandrock et al. (2014) was not reflecting field-realistic conditions, as colonies were artificially fed instead of foraging on treated crops. No evidence of adverse effects of neonicotinoid seed treatment is reported from monitoring studies (Fairbrother et al. 2014) and reviewed by (Schmuck and Lewis 2016). However, a correlative study claimed to have detected an increased risk for honey bee colonies to die in correlation with exposure to residues originating from seed treatment with imidacloprid (Budge et al. 2015). Further investigations may help to better judge the validity of such correlative approaches, which exclusively focus on one single potential causative factor.

Residues of the parent compound clothianidin and of its metabolites TZNG and TZMU were analyzed in stored syrup, in forager bees, hive bees, bee bread, and larvae. No accumulation was observed. During winter, residues in the stored syrup decreased to the half of the initial concentrations, but did not completely disappear. After winter, a maximum concentration of 28.8 µg kg^−1^ was found in stored syrup of C50 colonies, which, however, originated from an unrealistically high exposure level. On average the NOEC level of 10 µg L^−1^ syrup respective of 8.13 µg kg^−1^ (European Food Safety Authority (efsa) 2013) (see Table 1) was not exceeded in group C0 and C10. A critical point is the presence of residues of clothianidin (<1.7 µg kg^−1^) in syrup of the control C0 colonies. The origin of these residues is unclear. Possibly robbing bees could have taken away clothianidin-contaminated food from colonies of group C10 or C50 or C200. A larger distance between the colonies could have reduced the problem of robbery. We did not opt for a wide spacing of the experimental colonies. It was intended to perform the study on a single experimental site to reduce the impact of environmental factors on the experiment. Nevertheless, we think that the findings of the study are reliable. The samples of stored food of the C0 colonies had low level of residues or even levels below LOQ (16 out of 23 samples; see online resource). Comparing the different matrices stored syrup contained the highest concentrations of residues (mean 22.9 µg kg^−1^). Concentrations of residues and metabolites in living adult bees were low with a mean of 2.1 µg kg^−1^ for foragers and 7.1 kg^−1^ for hive bees. Low concentrations were measured in larvae (mean 2.7 µg kg^−1^*).*We can therefore conclude that the colonies were in fact exposed during the whole duration of the experiment. Regarding this point the experimental design was suitable to reveal possible effects. Obviously, no bee product acts as sink for clothianidin or its metabolites. There seems to be a dynamic equilibrium between the input of the substance, its metabolization and the elimination of the pesticide.

## Electronic supplementary material

Supplementary Materials
