# Supplementary material for: A long-term field study on the effects of dietary exposure of clothianidin to varroosis-weakened honey bee colonies
Source: Ecotoxicology. 2018 May 3;27(7):772–83. doi: 10.1007/s10646-018-1937-1 (PMC6133000; doi:10.1007/s10646-018-1937-1)
Supplement: Supplementary file 1 — Supplementary Materials [file 10646_2018_1937_MOESM1_ESM.docx]

Online Resource 1

Mean residues of clothianidin [µg kg^-1^], LOQ = 1 µg kg^-1^, LOD = 0.3 µg kg^-1^, Mean values were calculated from the values >LOQ.

| Date (Days after initiation) | group | Matrix | Mean^§^ | N^1^ | Minimum | Maximum | Number of samples below LOQ |
| --- | --- | --- | --- | --- | --- | --- | --- |
| 13.08.2014  69 DAI | C0 | Bee bread | <LOQ | 6 | <LOD | <LOQ | 6 |
|  |  | Forager bees | <LOD | 6 | <LOD | <LOD | 6 |
|  |  | Food | 1.4 | 6 | <LOQ | 1.9 | 1 |
|  |  | Hive bees | <LOQ | 6 | <LOD | <LOQ | 6 |
|  |  | Larvae | <LOD | 6 | <LOD | <LOD | 6 |
|  | C10 | Bee bread | 1.8 | 6 | 1.1 | 2.2 | 0 |
|  |  | Forager bees | <LOD | 6 | <LOD | <LOQ | 6 |
|  |  | Food | 7.3 | 6 | 6.8 | 8.1 | 0 |
|  |  | Hive bees | 2.4 | 6 | <LOQ | 3.1 | 1 |
|  |  | Larvae | 1,7 | 6 | <LOD | 1.7 | 5 |
|  | C50 | Bee bread | 8.1 | 6 | 5.4 | 12 | 0 |
|  |  | Forager bees | 1.3 | 6 | <LOQ | 1.3 | 5 |
|  |  | Food | 27.4 | 6 | 24 | 31 | 0 |
|  |  | Hive bees | 5.2 | 6 | 3.1 | 7.6 | 0 |
|  |  | Larvae | 1.7 | 6 | <LOQ | 3.0 | 1 |
|  | C200 | Bee bread |  | 0 |  |  |  |
|  |  | Forager bees |  | 0 |  |  |  |
|  |  | Food | 108.6 | 6 | 59.3 | 151.0 | 0 |
|  |  | Hive bees | 22.7 | 5 | 10.7 | 39.6 | 0 |
|  |  | Larvae | 8.2 | 2 | 7.8 | 8.7 | 0 |
| 25.09.2014  112 DAI | C0 | Bee bread | <LOD | 6 | <LOD | <LOQ | 6 |
|  |  | Forager bees | <LOD | 6 | <LOD | <LOD | 6 |
|  |  | Food | <LOD | 6 | <LOD | <LOQ | 6 |
|  |  | Hive bees | <LOD | 6 | <LOD | <LOD | 6 |
|  |  | Larvae | <LOD | 6 | <LOD | <LOD | 6 |
|  | C10 | Bee bread | <LOQ | 6 | <LOD | <LOQ | 6 |
|  |  | Forager bees | <LOD | 6 | <LOD | <LOQ | 6 |
|  |  | Food | 3.9 | 6 | <LOQ | 6.0 | 2 |
|  |  | Hive bees | <LOD | 6 | <LOD | <LOD | 6 |
|  |  | Larvae | <LOD | 6 | <LOD | <LOD | 6 |
|  | C50 | Bee bread | 1.9 | 6 | <LOQ | 2.6 | 1 |
|  |  | Forager bees | <LOD | 6 | <LOD | <LOQ | 6 |
|  |  | Food | 11.2 | 6 | 2.3 | 20 | 0 |
|  |  | Hive bees | 2.0 | 6 | <LOQ | 3.3 | 2 |
|  |  | Larvae | 1.2 | 5 | <LOQ | 1.4 | 2 |
| 18.03.2015  286 DAI | C0 | Bee bread | <LOD | 4 | <LOD | <LOD | 4 |
|  |  | Forager bees | <LOD | 4 | <LOD | <LOD | 4 |
|  |  | Food | 1.7 | 6 | <LOD | 2.0 | 4 |
|  |  | Hive bees | <LOD | 5 | <LOD | <LOD | 5 |
|  |  | Larvae | <LOD | 5 | <LOD | <LOD | 5 |
|  | C10 | Bee bread | 1.6 | 5 | <LOD | 1.7 | 2 |
|  |  | Forager bees | >LOQ | 3 | <LOD | <LOQ | 3 |
|  |  | Food | 4.2 | 5 | 2.4 | 5.8 | 0 |
|  |  | Hive bees | <LOQ | 3 | <LOD | <LOQ | 3 |
|  |  | Larvae | <LOD | 3 | <LOD | <LOD | 3 |
|  | C50 | Bee bread | 2.8 | 4 | 1.2 | 3.7 | 0 |
|  |  | Forager bees | 2.3 | 5 | 1.6 | 3.1 | 0 |
|  |  | Food | 22.8 | 5 | 17.7 | 26.9 | 0 |
|  |  | Hive bees | 2.5 | 5 | 1.2 | 3.7 | 0 |
|  |  | Larvae | 1.4 | 5 | <LOD | 1.4 | 4 |
| 22.04.2015  321 DAI | C0 | Bee bread | <LOD | 5 | <LOD | <LOD | 5 |
|  |  | Forager bees | <LOD | 5 | <LOD | <LOD | 5 |
|  |  | Food | <LOQ | 5 | <LOD | <LOQ | 5 |
|  |  | Hive bees | <LOD | 5 | <LOD | <LOD | 5 |
|  |  | Larvae | <LOD | 5 | <LOD | <LOD | 5 |
|  | C10 | Bee bread | 1.5 | 3 | <LOQ | 1.5 | 2 |
|  |  | Forager bees | <LOD | 3 | <LOD | <LOD | 3 |
|  |  | Food | 4.8 | 3 | 4.4 | 5.5 | 0 |
|  |  | Hive bees | <LOD | 3 | <LOD | <LOD | 3 |
|  |  | Larvae | <LOD | 3 | <LOD | <LOD | 3 |
|  | C50 | Bee bread | 4.7 | 5 | 1.8 | 6.7 | 0 |
|  |  | Forager bees | <LOQ | 5 | <LOD | <LOQ | 5 |
|  |  | Food | 22.7 | 5 | 16.1 | 28.8 | 0 |
|  |  | Hive bees | <LOQ | 5 | <LOD | <LOQ | 5 |
|  |  | Larvae | <LOQ | 5 | <LOD | <LOQ | 5 |
|  |  |  |  | N_obs_ |  |  |  |
| Overall |  | Bee bread | 3.6 | 62 | <LOD | 12.21 | 32 |
|  |  | Forager bees | 2.1 | 61 | <LOD | 3.1 | 55 |
|  |  | Food | 22.9 | 71 | <LOD | 151.1 | 18 |
|  |  | Hive bees | 7.1 | 67 | <LOD | 39.6 | 42 |
|  |  | Larvae | 2.7 | 63 | <LOD | 8.7 | 51 |
|  |  | 69 DAI | 18.5 | 103 | <LOD | 151.0 | 44 |
|  |  | 112 DAI | 4.7 | 89 | <LOD | 20.4 | 67 |
|  |  | 286 DAI | 6.0 | 67 | <LOD | 27.1 | 38 |
|  |  | 321 DAI | 11.0 | 65 | <LOD | 28.8 | 51 |

^§^ Means for detections > LOQ;  ^1^ number of tested colonies; N_obs_: Number of samples
